# Local Control of Distal Cholangiocarcinoma With Radiofrequency Ablation at Endoscopic Retrograde Cholangiopancreatography

**DOI:** 10.14309/crj.0000000000001382

**Published:** 2024-06-17

**Authors:** Lizeth Cifuentes, Charles Gabbert, Adam Slivka

**Affiliations:** 1 Department of Medicine, University of Pittsburgh Medical Center, Pittsburgh, PA; 2 Division of Gastroenterology & Hepatology, University of Pittsburgh Medical Center, Pittsburgh, PA

**Keywords:** distal cholangiocarcinoma, endoscopic approach, radiofrequency ablation

## Abstract

Distal cholangiocarcinoma (CCA) can pose diagnostic and therapeutic challenges, often leading to a poor prognosis. While curative resection is viable for a minority in the early stage, we report a case of successful endoscopic therapy. A 79-year-old patient, diagnosed with early-stage extrahepatic CCA, opted out of surgery and chemotherapy. Instead, he pursued a treatment strategy involving serial radiofrequency ablation with stent exchange at endoscopic retrograde cholangiopancreatography. The patient achieved remission, showcasing the potential for local control of distal CCA through radiofrequency ablation and covered self-expanding metal stents. This alternative becomes particularly relevant for patients unsuitable for surgery or chemotherapy and those who decline it.

## INTRODUCTION

Distal cholangiocarcinoma (CCA), a rare and highly aggressive malignant tumor emerging from the bile duct epithelium between the origin of the cystic duct and the ampulla of Vater, poses significant challenges in diagnosis and treatment. The limited success of curative resection, primarily through pancreaticoduodenectomy, is reserved for a minority diagnosed at an early stage. Statistics reveal a 5-year survival rate of only 27% after resection with negative margins, underscoring the formidable nature of this malignancy.^1^ Compounding the issue, a majority of explored tumors prove nonresectable.^2^ Amidst this challenging landscape, this case presents an endoscopic approach involving serial radiofrequency ablation (RFA) with stent exchange demonstrating success in managing distal CCA.

## CASE REPORT

**Figure 1.**
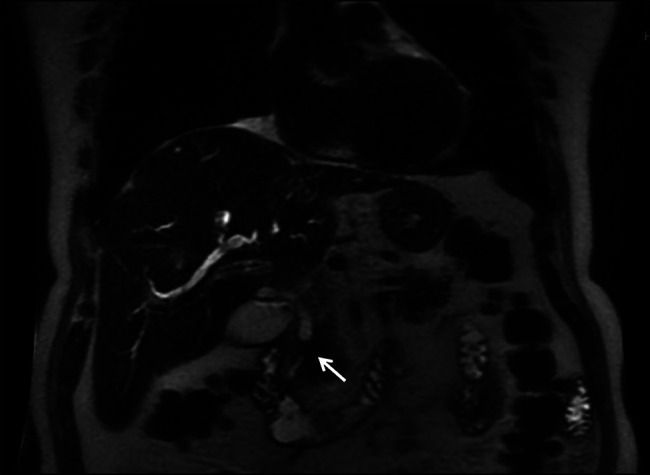
Abdominal magnetic resonance imaging showing moderate intrahepatic and extrahepatic biliary ductal dilatation with the extrahepatic common bile duct measuring 11 mm in the region of the porta hepatis and a 1 cm short-segment stricture (white arrow) involving the distal common bile.

A 79-year-old man with a medical history of obesity class 2, hypertension, hyperlipidemia, alongside a history of prostate cancer managed with prostatectomy, presented for his annual wellness examination. Unremarkable for abdominal pain, the physical examination unveiled mild jaundice, prompting further investigation. Blood work indicated a cholestatic liver injury, prompting an abdominal ultrasound that unveiled a dilated common bile duct with mild intrahepatic dilation. Subsequent magnetic resonance imaging uncovered a 1 cm short-segment stricture in the distal common bile duct without an associated pancreatic mass or metastatic disease (Figure 1). An endoscopic retrograde cholangiopancreatography (ERCP) revealed a tight distal bile duct stricture. Tissue sampling during the ERCP unveiled atypical cells positive for neoplastic changes, signaling at least high-grade dysplasia. Next-generation sequencing then confirmed the diagnosis of CCA, exposing kRAS, p53, and SF3B7 mutations.^3^

A plastic biliary stent was placed, and the patient was referred to surgical oncology for further management. The patient opted against a recommended pancreaticoduodenectomy and also refused radiation or chemotherapy, opting instead for palliative treatment with biliary stents.

**Figure 2.**
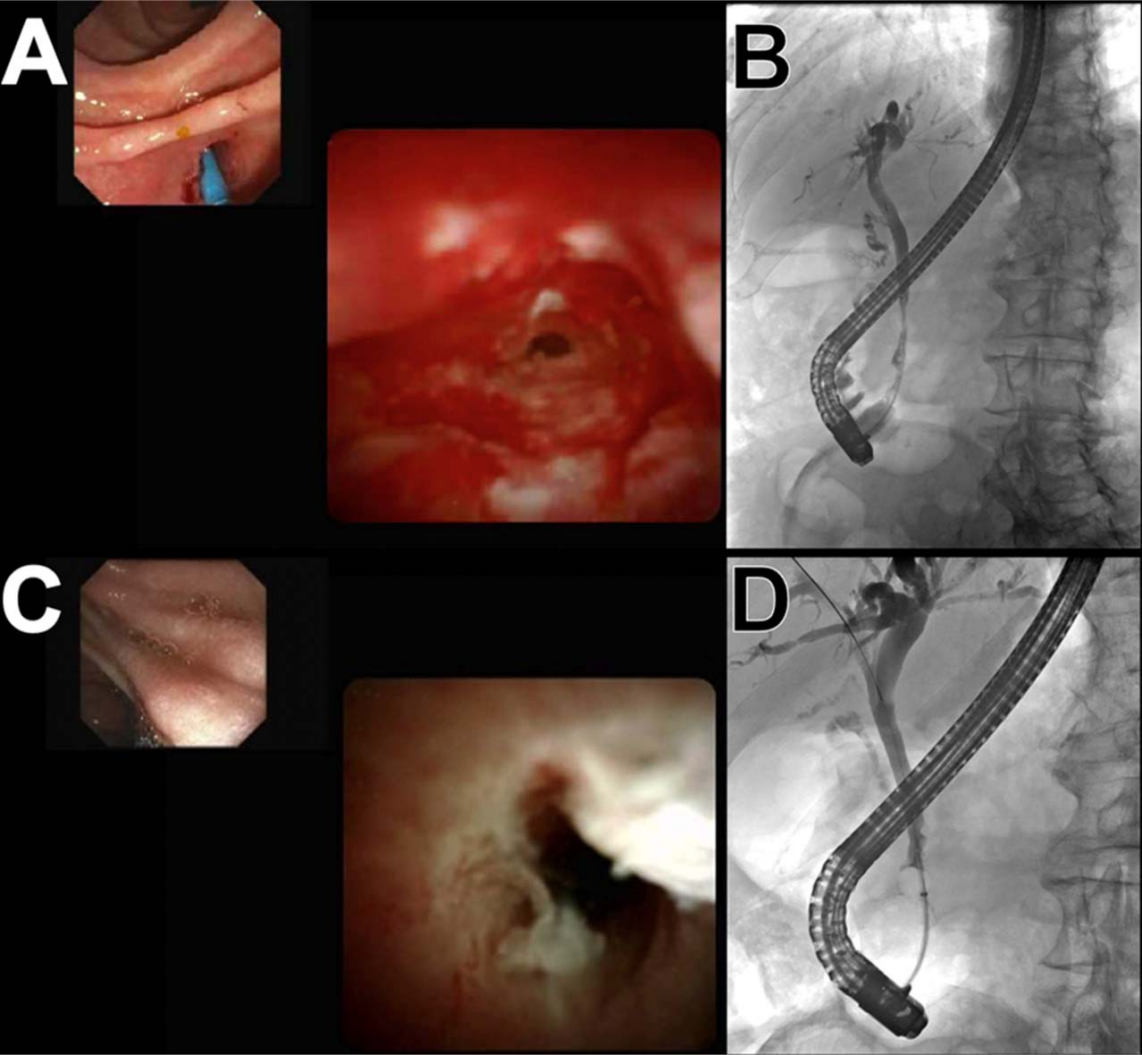
(A) Cholangioscopic view, and (B) Cholangiogram of the distal stricture before radiofrequency ablation (RFA). (C) Cholangioscopic view, and (D) Cholangiogram, both showcasing the nearly complete resolution of the previously observed distal common bile duct stricture following 2 rounds of RFA.

A decision was made to pursue local control through a combination of RFA and the placement of covered metal stents (cSEMSs) during serial ERCP procedures every 6 to 8 months. RFA was applied using a 22 mm probe at 10 W for 2 min (ELRA; Tae-Woong Medical, Los Angeles, CA). Following the third session, cholangioscopy revealed resolution of the previously seen stricture in the distal common bile duct. Two years after index presentation, a computed tomography scan showed no signs of mass or metastatic disease. Subsequent cholangioscopy disclosed scar tissue and minimal nodularity around the prior stricture, which was biopsied under direct visualization showing only granulation tissue on histology and no mutations on next-generation sequencing (Figure 2).

## DISCUSSION

This case highlights the potential for achieving local control of extrahepatic distal CCA through the implementation of RFA and cSEMS. RFA, leveraging a high-frequency alternating current to induce thermal damage in tissue,^4^ is known for its efficacy in treating solid liver tumors, particularly in hepatocellular cancer.^5^ However, the applicability of percutaneous RFA to CCA is hindered by poor visualization and the risk of damaging adjacent structures.

RFA has emerged as a modality to achieve some local control of CCA during ERCP by using an over-the-wire endoluminal biliary catheter.^6^ This approach facilitates precise delivery of thermal energy to the malignant stricture. The extent of penetration achieved in RFA for CCA varies based on the specific methodology and tumor dimensions. In 2018, a study revealed a median maximal ablation depth of 4.0 mm in distal extrahepatic CCA.^7^ Another investigation, notated for its focus on intrahepatic CCA lesions with a size of ≤3.4 cm, reported technical effectiveness.^8^ In addition, 2 separate studies emphasized the potential efficacy of endoscopic intraductal RFA and percutaneous ultrasound-guided RFA for CCA treatment.^9,10^ Despite these findings, further investigations are required to ascertain the optimal penetration depth for diverse types and stages of CCA.

RFA has become more frequently considered as a locoregional therapy in selected patients with intrahepatic CCA when surgery is not an option with an increased in 5-year survival rates on observational studies.^11^ However, RFA is less frequently used in extrahepatic CCA only. Concurrently, cSEMS, a preferable choice over plastic stents for palliative treatment in malignant distal biliary obstruction, offer extended stent patency, reduced need for reintervention, enhanced survival rates, and achieved notable cost-effectiveness.^12^ Covered stents may induce some pressure necrosis within the treated stricture and offer the advantage of being removable. This integrated approach showcases the potential of RFA and cSEMS in navigating the complexities of distal CCA, providing a viable alternative for patients who may be unsuitable for surgery or opt to decline it.

Fortunately, our patient did not encounter any adverse effects; however, it is crucial to acknowledge potential complications associated with RFA. These complications typically arise from the thermal effect generated during the procedure, among them flu-like syndrome, pain, and thermal injury to adjacent organs. In the realm of pancreatobiliary RFA, the most concerning adverse events encompass pancreatitis, bile duct strictures, pleural effusions, biliary fistulas, cholangitis, cholecystitis, and bleeding.^13^ While our case underscores the success of the chosen intervention, understanding and vigilance regarding potential complications are crucial for the comprehensive management of patients undergoing such procedures.

The long-term durability of the complete response observed in this patient will require continued follow-up. However, this approach offers a promising alternative for patients who are not suitable candidates for surgery or those who decline it. RFA has already proven its value in the local control of patients with hilar CCA^14^ and should be considered an essential tool in the arsenal of endoscopists treating all forms of extrahepatic CCA.

## DISCLOSURES

Author contributions: All authors contributed to the concept, design, drafting, revision, and final approval of the article. L. Cifuentes is the article guarantor.

Financial disclosure: None to report.

Informed consent was obtained for this case report.
